# Combined estrogenic and anti-estrogenic properties of estetrol on breast cancer may provide a safe therapeutic window for the treatment of menopausal symptoms

**DOI:** 10.18632/oncotarget.4184

**Published:** 2015-05-19

**Authors:** Céline Gérard, Mélanie Mestdagt, Ekaterine Tskitishvili, Laudine Communal, Anne Gompel, Elisabete Silva, Jean-François Arnal, Françoise Lenfant, Agnès Noel, Jean-Michel Foidart, Christel Péqueux

**Affiliations:** ^1^ Laboratory of Tumor and Development Biology, GIGA-Cancer, University of Liège, Liège, Belgium; ^2^ Gynaecological Endocrinology Unit, Paris Descartes University, Hôpitaux Universitaires, Paris, France; ^3^ INSERM U 938, UPMC, Paris; ^4^ Institute of Environment, Health and Societies, Brunel University London, UB83PH Uxbridge, United Kingdom; ^5^ INSERM U1048, Institut des Maladies Métaboliques et Cardiovasculaires, University of Toulouse, UPS, Toulouse, France

**Keywords:** estetrol, menopause, breast cancer, estrogen receptor, SERM

## Abstract

Increased risk of breast cancer is a critical side effect associated with the use of a menopausal hormone therapy (MHT). Estetrol (E4) is a natural estrogen produced by the human fetal liver and is a promising compound for clinical use in MHT. However, its impact on breast cancer is controversial and poorly defined. In this preclinical study, we show that E4 acts as a weak estrogen by stimulating the growth of hormone-dependent breast cancer only at concentrations exceeding menopausal therapeutic needs. E4 presents also an antitumor activity by decreasing the strong proliferative effect of estradiol (E2). While estrogen receptor alpha (ERα) is the predominant receptor mediating its effects, the dual weak-estrogenic/anti-estrogenic feature of E4 results from differential signaling pathways activation. Both nuclear and rapid extra-nuclear signaling pathway are necessary for a complete estrogenic effect of E4. However, the antitumor action of E4 is not due to a capacity to antagonize E2-induced nuclear activity. Altogether, our results highlight that E4 has a limited impact on breast cancer and may offer a safe therapeutic window for the treatment of menopausal symptoms.

## INTRODUCTION

Menopausal complaints such as hot flushes, sexual and sleep disorders or loss of bone mineral density are usually relieved by the administration of an estrogen [1]. However, menopausal hormone therapy (MHT) has been associated with severe side effects. Due to their strong hepatic impact, estrogens increase the incidence of thromboembolic events [2-4]. Moreover the administration of an estrogen combined with progestin increases the risk of breast cancer [3, 5-7]. These observations highlight the need to develop new MHT with safer compounds that retain the beneficial effects of estrogens on the bone, uro-genital and central nervous systems, while exhibiting minimal impact on hepatic and mammary tissues.

Estetrol (E4) is a natural estrogen exclusively produced by the human fetal liver during pregnancy [8]. Several animal and *in vitro* studies indicate that E4 presents a biological profile similar to selective estrogen receptor modulators (SERMs). It exhibits estrogen-like effects on the brain [9-11], bone [12], uterus [13-15], ovulation [16] and atheroma prevention [13]. E4 also presents anti-estrogenic properties in vascular and central nervous systems since it prevents E2 actions on endothelial NO synthase activation, acceleration of endothelial healing and on allopregnanolone synthesis [10, 13]. In addition, E4 decreases the proliferative effect of E2 on normal breast epithelial cells [17]. E4 has a considerably lower impact on coagulation and hemostasis than ethinylestradiol (EE) or estradiol (E2) and might therefore have a minimal impact on the incidence of thromboembolic events (Foidart, Congress of Eur. Soc. Gynecol. 2013, personal communication).

These unique pharmacological properties support that E4 is a suitable and safe compound for MHT. However, the impact of E4 on breast cancer is only partially documented, poorly understood and controversial. It has been reported that E4 prevents and suppresses mammary tumor growth in rats treated with DMBA (7,12dimethylbenz(a)anthracene) as efficiently as ovariectomy or tamoxifen [18]. However, *in vitro* studies conducted in ER-positive breast cancer cell lines revealed that E4 promotes cell growth [19, 20]. In a clinical study of women with ER-positive breast cancer, it was recently observed that E4 was pro-apoptotic but did not decrease the expression of the proliferation marker Ki-67 in tumor cells [21]. Finally, Giretti [22] reported that E4 moderately stimulates breast cell migration and invasion but is also able to antagonize the effect of E2 on these processes.

Seventy percent of breast tumors express the estrogen receptor alpha (ERα). In these cancers, E2 acts as a growth factor promoting tumor growth through a highly complex and not fully understood signaling system. Most of the actions of E2 are mediated by nuclear receptors ERα and ERβ [23]. In a classical way, after dimerization and co-regulators recruitment, the E2/ER complexes can directly bind estrogen responsive element (ERE) in the promoter region of target genes to modulate their expression. ER can also act as co-activators for other transcription factors such as AP-1 and Sp1 that mediate the transcription of genes whose promoters do not harbor ERE [24, 25]. Besides these nuclear effects, E2 also elicits extra-nuclear actions related to rapid signaling pathway activation and commonly referred as membrane initiated steroid signaling (MISS) [26-28]. These effects are mediated through a pool of membrane-anchored ERα. Another putative estrogen membrane receptor termed G protein-coupled estrogen receptor 1 (GPER), also referred as GPR30, has been proposed to contribute to physiological and tumor promoting effects of estrogens [29].

The aim of this study is to define the impact of E4 on breast cancer growth. We investigated its effect on several *in vitro* and *in vivo* breast cancer models using a large range of concentrations. Special attention was devoted on molecular mechanisms driving E4 effects.

## RESULTS

### E4 is weaker than E2 to increase the growth of ERα-positive breast cancer cells

**Figure 1:**
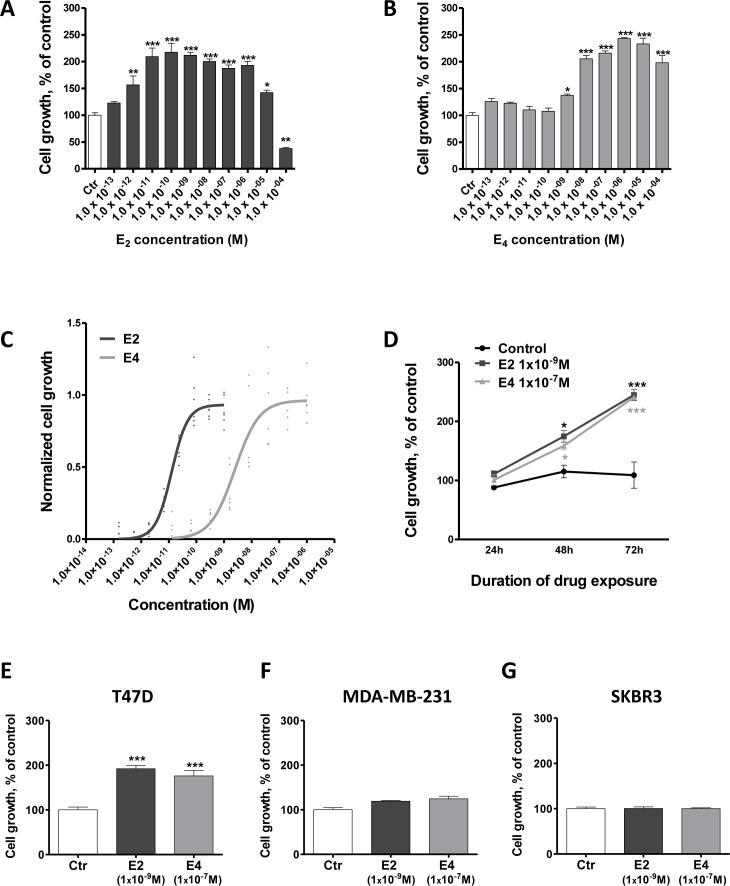
E4 is weaker than E2 in promoting the growth of breast cancer cells **A.-B.**, dose-dependent effect of E2 (A) and E4 (B) on the growth of MCF-7 cells. Cells were treated for 72 hours with vehicle (ctr) or increasing concentrations of E2 or E4. **C.**, effect of E2 and E4 in the E-Screen assay. MCF-7 BOS cells were treated with increasing concentrations of E4 (grey curve) or E2 (black curve). Graph represents three independent experiments run in duplicate and shows experimental data (dots) with best fit regression curves. **D.**, time-course experiment. MCF-7 cells were cultured for up to 3 days in presence of E2 1×10^−9^M (square), E4 1×10^−7^M (triangle) or vehicle (control, circle). **E.-G.**, growth of T47D (E), MDA-MB-231 (F) and SKBR3 (G) cells treated with vehicle (ctr), E2 1×10^−9^M or E4 1×10^−7^M. Data are represented as mean ± SEM (*n* = 5). *: *P*≤0.05; **: *P* ≤ 0.01; ***: *P* ≤ 0.001 versus control group.

We first compared the effect of E2 and E4 on the growth of MCF-7 breast cancer cells, which express ERα, ERβ and GPER (Suppl. Figure 1). E2 tested at concentrations ranging from 1×10^−13^ to 1×10^−4^M induced a typical bell shaped dose-response curve, with a maximal response of 2.2-fold reached at 1×10^−11^M (Figure 1A). High concentrations of E2 (≥1×10^−5^M) caused a rapid decline in cell growth, indicative of cytotoxicity. At 1×10^−11^M, E4 did not increase MCF-7 cell growth (Figure 1B), but a 1,000-times higher concentration (1×10^−8^M) was necessary to promote growth to the same extent than E2.

Dose-response curves for E2 and E4 recorded in the E-Screen assay (Figure 1C), also showed that E4 produced a weak agonistic activity compared to E2 with an EC50 of 4×10^−9^M and a maximal mitogenic effect around 1×10^−7^M, demonstrating a 200 times weaker capacity to induce MCF-7 BOS cell growth than E2 (EC50 = 2×10^−11^M).

When used at a stimulating concentration (E2 1×10^−9^M and E4 1×10^−7^M), both compounds increased MCF-7 cell growth in a similar time-dependent manner (Figure 1D). It is noteworthy to consider that such E4 pharmacological high doses would never be used in clinical setting.

We then evaluated the impact of E4 on breast cancer cell lines exhibiting different pattern of estrogen receptor expression (Suppl. Figure 1): T47D (ERα+, ERβ−, GPER+), MDA-MB-231 (ERα−, ERβ+, GPER+) and SKBR3 (ERα−, ERβ−, GPER+) cells. After 72h of treatment, E2 (1×10^−9^M) and E4 (1×10^−7^M) increased the growth of T47D cells similarly to what was seen for MCF-7 cells (Figure 1E). By contrast, E2 and E4 failed to stimulate the growth of MDA-MB-231 (Figure 1F) and SKBR3 cells (Figure 1G).

Altogether, these data support that E4 increases cell growth only at high concentrations and that ERα expression is required for this mitogenic activity.

### E4 increases cell growth by stimulating proliferation and preventing cell death

**Figure 2:**
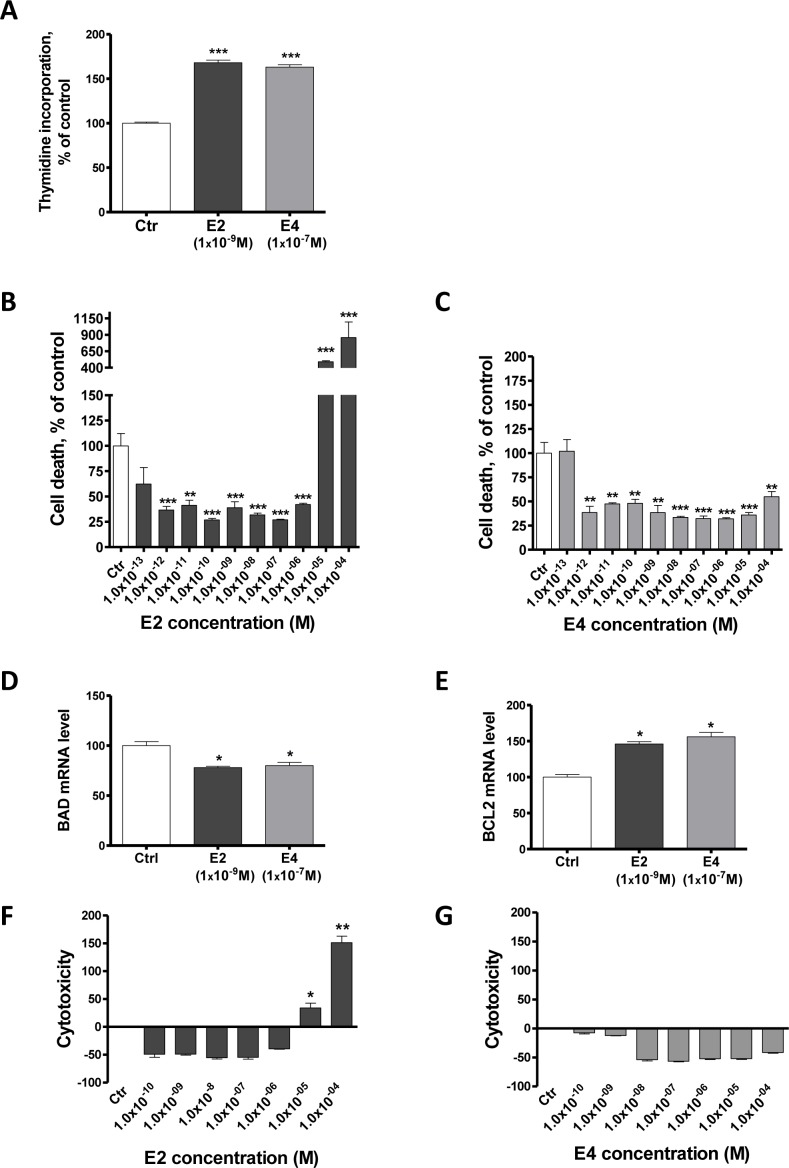
E4 increases cell proliferation and reduces apoptosis of breast cancer cells **A.**, proliferation of MCF-7 cells treated with vehicle (ctr), E2 1×10^−9^M or E4 1×10^−7^M. Proliferation was evaluated using a thymidine incorporation assay after 72 hours of treatment. **B.**-**C.**, Cell death level in MCF-7 cells treated for 72 hours with vehicle (ctr) or increasing concentrations of E2 (B) or E4 (C). **D.**-**E.**, quantitative RT-PCR analysis of pro-apoptotic gene *BAD* (D) and anti-apoptotic gene *BCL2* (E) from MCF-7 cells treated with vehicle (ctrl), E2 1×10^−9^M or E4 1×10^−7^M during 6 hours. **F.**-**G.**, cytotoxicity induced by increasing concentrations of E2 (F) or E4 (G) in MCF-7 cells after 72 hours of treatment. Data are represented as mean ± SEM (*n* = 3). *: *P* ≤ 0.05; **: *P* ≤ 0.01; ***: *P* ≤ 0.001 versus control group.

Estrogens stimulate cell growth in ERα-positive cells by increasing cell proliferation and reducing apoptosis [30]. To determine the impact of E4 on proliferation and cell death, a thymidine incorporation assay and a quantification of histone-complex DNA fragments were carried out on MCF-7 cells. As expected, E2 stimulated the proliferation of MCF7 cells (Figure 2A). A 100 times higher concentration of E4 was needed to achieve the same effect. Cells treated with E2 ≤ 1×10^−6^M exhibited a ~ 60% lower level of cell death than control cells but at concentrations > 1×10^−6^M, E2 dramatically stimulated cell death (Figure 2B). In a similar way to E2, E4 reduced the cell death level in treated cells (Figure 2C). Interestingly, in contrast to E2, E4 did not induce cell death when used at high concentrations. In addition, both E4 (1×10^−7^M) and E2 (1×10^−9^M) significantly decreased the expression of the pro-apoptotic gene BAD (Figure 2D) and up-regulated the anti-apoptotic gene BCL2 (Figure 2E), supporting an impact on apoptosis..

A cytotoxicity test revealed that E2 concentrations ranging from 1×10^−10^M to 1×10^−6^M decreased MCF-7 cytotoxicity, but that E2 was concentrations of 1×10^−5^M and 1×10^−4^M were cytotoxic (Figure 2F). E4 did not show any sign of cytotoxicity for concentrations up to 1×10^−4^M (Figure 2G).

### E4 increases cell growth primarily via ERα

**Figure 3:**
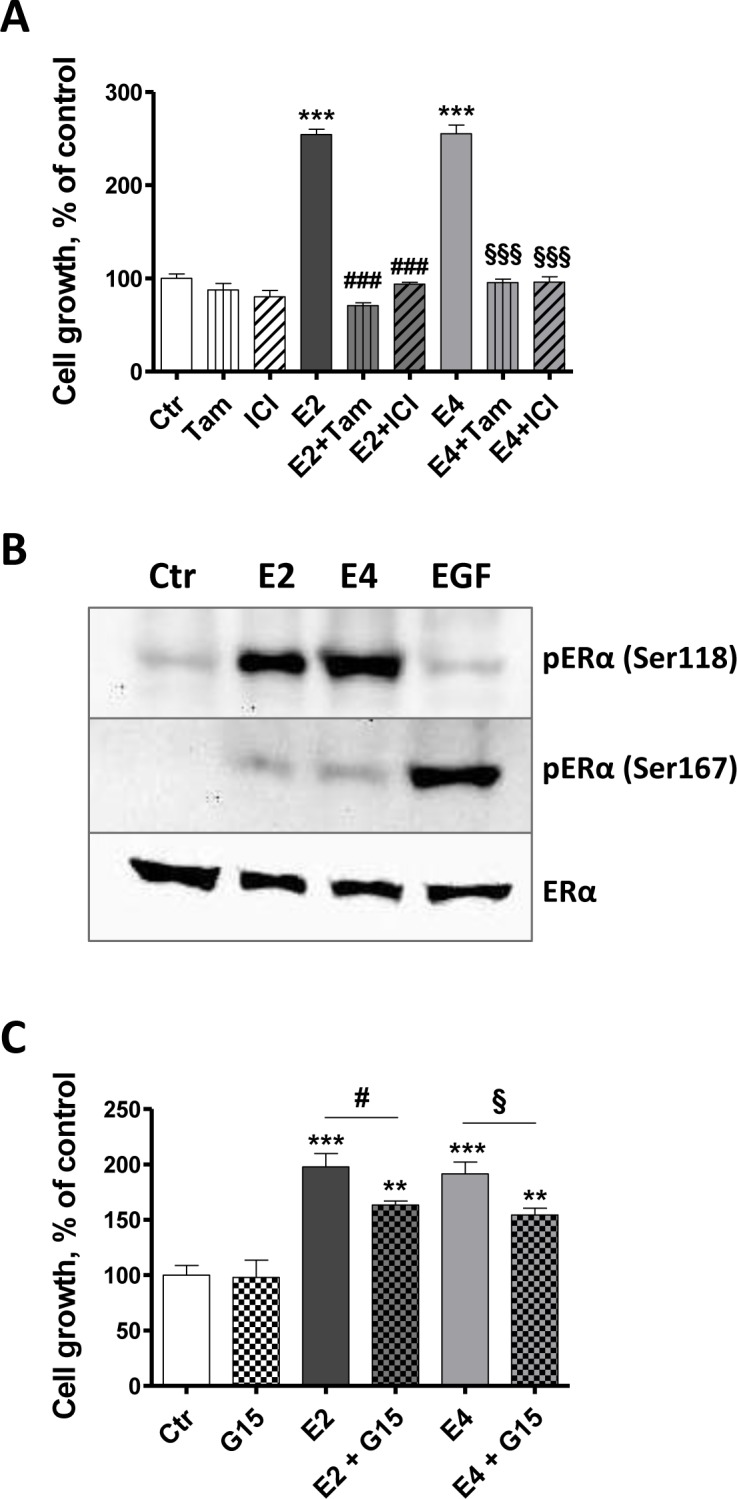
E4 stimulates cell growth primarily via ERα **A.**, MCF-7 cell growth in the presence of antiestrogens fulvestrant and tamoxifen. Cells were exposed to vehicle (ctr), 1×10^−7^M fulvestrant (ICI), 1×10^−7^M tamoxifen (tam), 1×10^−9^M E2, 1×10^−7^M E4 or combination of estrogens and antiestrogens. **B.**, representative western blot of estrogen receptor alpha phosphorylation after a treatment of 30 minutes with vehicle (ctr), E2 1×10^−9^M, E4 1×10^−7^M or EGF 100 ng/ml. **C.**, MCF-7 cell growth in the presence of G15 1×10^−6^M, a GPER antagonist. Cell growth was evaluated after 72 hours of treatment. Data are represented as mean ± SEM (*n* = 5). *, *P* ≤ 0.05; **, *P* ≤ 0.01; ***, *P* ≤ 0.001 versus control group. ♯, *P* ≤ 0.05; ♯♯♯, *P* ≤ 0.001 versus E2 group. §, *P* ≤ 0.05; §§§, *P* ≤ 0.001 versus E4 group.

Using tamoxifen, an ERα antagonist in the breast, and ICI 182 780, a pure ERα antagonist, we observed that the combinations of E4 with both antiestrogens decreased MCF-7 cell growth to level of untreated cells (Figure 3A). This observation confirms that the stimulating effect of E4 is mediated by ERα. ERα is described to be activated by phosphorylation on key serine residues among which Ser118, the major phosphorylation site when the receptor is activated by estrogens, and Ser167 phosphorylated through the EGF/EGFR pathways [31]. The stimulation of MCF-7 cells with E2 (1×10^−9^M) or E4 (1×10^−7^M) led to a robust phosphorylation of Ser118 and to a weak phosphorylation of Ser167 (Figure 3B), highlighting the activation of ERα by these compounds. While E4 was unable to stimulate the proliferation of SKBR3 cells which express GPER but not ERα, G15, a reported GPER antagonist, partially decreased the E2- and E4-induced MCF-7 cell growth (Figure 3C).

These data confirm our above observations (Figure 1) that ERα is the predominant receptor mediating the mitogenic effects of E4. They also suggest a possible contribution of GPER to this process. It has been indeed suggested that GPER may facilitate membrane-initiated steroid signaling under limited circumstances [32].

### E4 elicits estrogenic effect by increasing transcriptional activity of ERα

**Figure 4:**
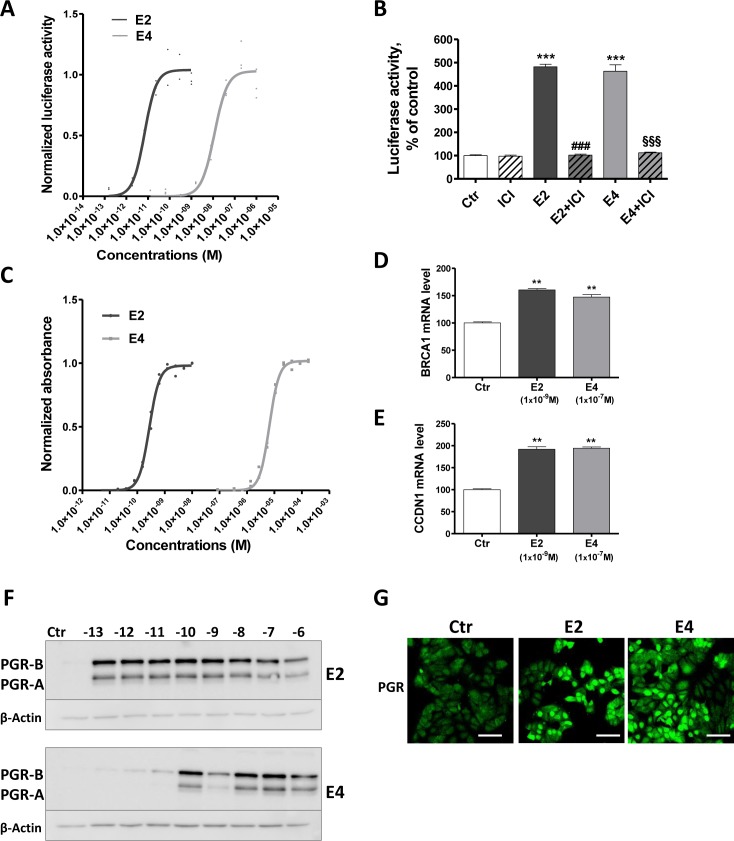
E4 enhances transcriptional activity of ERα **A.**, normalized effect of increasing concentrations of E2 (black curve) and E4 (grey curve) on ERE-luciferase reporter activity in T47D-KBluc cells. **B.**, normalized luciferase activity of T47D-KBluc cells treated with vehicle (ctr), ICI 182,780 (1×10^−7^M), E2 (1×10^−9^M), E4 (1×10^−7^M) or a combination of estrogen and ICI 182,780. ERE-luciferase reporter activity was measured after 24 hours. **C.**, normalized effect of increasing concentrations of E2 (black curve) and E4 (grey curve) on absorbance in the recombinant yeast estrogen screen. Graph shows experimental data (dots) with best fit regression curves. **D.**-**E.**, quantitative RT-PCR analysis of *BRCA1* (D) and *CCDN1* (E) genes from MCF-7 cells treated with vehicle (ctr), E2 1×10^−9^M or E4 1x 10^−7^M during 6 hours. **F.**-**G.**, progesterone receptor (PGR) induction highlighted by western blot (F) in MCF-7 cells treated with increasing concentrations of E2 or E4 (from 1×10^−13^ to 1×10^−6^M) and by immonufluochemistry (G) after treatment with vehicle (ctr), E2 1×10^−9^M or E4 1×10^−7^M during 48 hours. Scale bar = 50μm. Data are represented as mean ± SEM. **, *P* ≤ 0.01; ***, *P* ≤ 0.001 versus control group. ♯♯♯, *P* ≤ 0.001 versus E2 group. §§§, *P* ≤ 0.001 versus E4 group.

To assess the impact of E4 on the activation and binding of ERα to ERE, we performed a luciferase reporter gene assay based on T47D-KBluc cells in presence of increasing concentrations of E2 or E4. As expected, E2 induced a strong ERE-Luc transactivation in T47D cells with an EC50 value of 1×10^−11^M. E4 also stimulated ERE-Luc transactivation, but the curve was shifted to higher concentrations, with an EC50 of 1×10^−8^M, and a maximal effect reached at 1×10^−7^M (Figure 4A). Similar results were obtained by using MCF-7 cells (Suppl. Figure 2). This E4-dependent transcriptional activity was completely abrogated by the ERα antagonist ICI 182,780 (Figure 4B), validating the specificity of the above results.

The weaker capacity of E4 to induce the binding of ERα to ERE was confirmed in a second model: the Yeast Estrogen Screen (Figure 4C), which is based on the ability of a compound to stimulate the expression of β-galactosidase in yeast by activating ERα. The normalized data showed that E4 had a potency of approximately 10,000 times less than E2 to activate the receptor and promote β-galactosidase production in the yeast.

We next investigated the capacity of E4 to promote non-classical nuclear effects by assessing the expression of genes that do not harbor ERE in their promoter region: *BRCA1* and *CCDN1*. These genes are thought to be regulated by the recruitment of ERα to AP-1 or Sp1 sites in the promoter region [33, 34]. Following 6 hours of incubation, both E4 (1×10^−7^M) and E2 (1×10^−9^M) up-regulated *BRCA1* and *CCDN1* mRNA (Figure 4D-4E).

Finally, to further demonstrate the transcriptional activation of ERα, we evaluated the induction of the progesterone receptor (PGR) protein, a well described estrogen-dependent process [35]. E4 led to a significant up-regulation of PGR in MCF-7 cells. At least 1×10^−10^M of E4 was required while E2 achieved a maximal effect at a concentration as low as 1×10^−13^M (Figure 4F). The induction of PGR in MCF-7 cells by E2 (1×10^−9^M) and E4 (1×10^−7^M) was also highlighted by immunofluochemistry (Figure 4G).

Altogether these data support that E4 is a weak inducer of ERα transcriptional activity by activating both classical and non-classical nuclear effects with a lower efficiency than E2.

### E4 activates extra-nuclear signaling cascades in breast cancer cells

**Figure 5:**
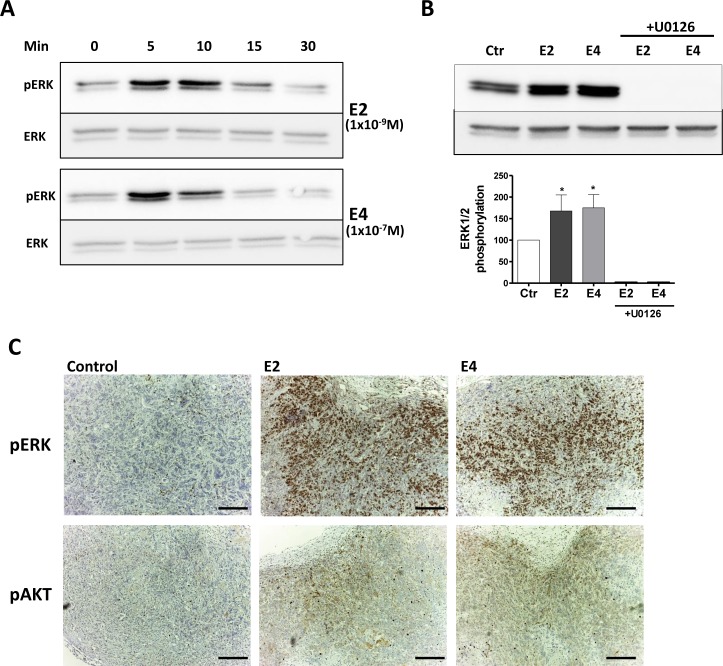
E4 activates extra-nuclear signaling pathways **A.**, representative western blot of ERK1/2 phosphorylation in MCF-7 cells treated with 1×10^−9^M E2 or 1×10^−7^M E4 during 5, 10, 15 and 30 minutes. **B.**, representative western blot and quantitative analysis of ERK1/2 phosphorylation in MCF-7 cells treated with vehicle (ctr), 1×10^−9^M E2, 1×10^−7^M E4 or combination of estrogens and 1×10^−5^M U0126 during 5 minutes. All treatments were performed in the presence of a same concentration of vehicle (ethanol 0,1%) **C.**, IHC staining of pERK and pAKT on MCF-7 tumors collected from mice treated with vehicle (control), E2 (provided by pellet) or E4 (3mg/kg/day) during 5 weeks. *, *P* ≤ 0.05 versus control group. Scale bar =200 μM.

The extra-nuclear effects of E2 on breast cancer rely on the activation of MAPK and PI3K/AKT pathways [36, 37]. Like E2 (1×10^−9^M), E4 (1×10^−7^M) increased the phosphorylation of ERK1/2 in a fast and transient manner, with a maximal activation seen after 5 min followed by a decline within 10 minutes (Figure 5A). U0126, an inhibitor of the upstream MEK1/2, completely abolished ERK1/2 phosphorylation attesting that the observed phosphorylation was induced by MEK in MCF-7 cells (Figure 5B). We also observed an increase in ERK1/2 and AKT phosphorylation *in vivo* in MCF-7 tumors resected from mice treated with E4 or E2 during 5 weeks (Figure 5C).

These results support that, in addition to nuclear effects, E4 is also able to activate extra-nuclear ERK1/2 and PI3K/AKT pathways.

### E4 increases breast tumor growth *in vivo* only at high concentrations, and antagonizes E2-dependent effect

**Figure 6:**
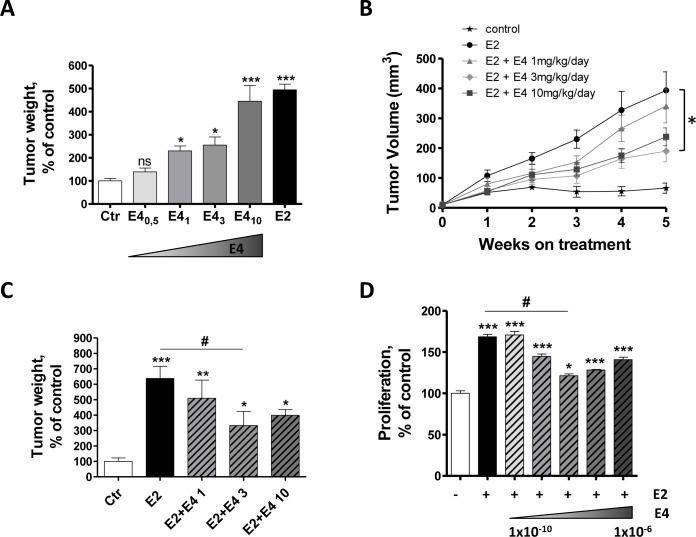
E4 moderately increases tumor growth *in vivo*, but antagonizes E2-dependent effect **A.**, Normalized weight of MCF-7 tumors resected from immunodeficient mice after 5 weeks of treatment. Mice were treated orally with vehicle (ctr), E2 (3mg/kg/day) or E4 (0.5 → 10 mg/kg/day). **B.**, *In vivo* growth curve of MCF-7 tumors in immunodeficient mice treated with vehicle (control, stars), estradiol only (E2, circle) or with a combination of E2 and E4 1 (triangle), 3 (diamond) or 10 (square) mg/kg/day during 5 weeks. **C.**, normalized weight of MCF-7 tumors collected from nude mice after 5 weeks of treatment with E2 alone or combinations of E2 and E4. **D.**, proliferation of MCF-7 cells treated with vehicle, E2 1×10^−10^M alone or with different combinations of E2 and E4. Increasing concentrations of E4 (from 1×10^−10^ to 1×10^−6^M) were added to a fixed concentration of E2 (1×10^−10^M) and proliferation was evaluated with a thymidine incorporation assay after 72 hours of treatment. Data are represented as mean ± SEM (*n* = 5). *, *P* ≤ 0.05; **, *P* ≤ 0.01; ***, *P* ≤ 0.001 versus control group. ♯, *P* ≤ 0.05 versus E2 group.

An estrogen supplementation is necessary for the growth of MCF-7 and the formation of tumor *in vivo*. To determine if E4 could achieve the same effect than E2, ovariectomized immunodeficient mice implanted with MCF-7 cells received a daily oral treatment of E4 (0.5, 1, 3 or 10 mg/kg/day) or E2 (3 mg/kg/day). After 5 weeks of treatment, E2 promoted tumor growth and tumor weights were 5-fold increased compared to the untreated group (Figure 6A). However, no significant difference was observed between untreated control group and mice treated with E4 0.5 mg/kg/day. E4 was as efficient as E2 in promoting tumor growth only at the dose of 10 mg/kg/day.

We then analyzed the effect of a combined treatment of E2 and E4 on MCF-7 tumor growth. Ovariectomized mice implanted with MCF-7 cells and with an E2 pellet received a daily oral treatment of E4 (1, 3 or 10mg/kg/day) during 5 weeks (Figure 6B). In these conditions, E4 antagonized E2-induced tumor growth in a dose-dependent manner. Exposure to the combination E2+E4 decreased the tumor volume and tumor weight by ~50% compared to mice exposed to E2 alone (Figure 6B-6C). This partial anti-estrogenic effect of E4 in the presence of E2 was also observed *in vitro* on MCF-7 cell proliferation. This effect became maximal when E4 was at least 100 times more concentrated than E2 (Figure 6D).

Altogether, these results indicate that E4 used alone acts as a weak estrogen and stimulates the growth of MCF-7 tumor *in vivo* only at high concentrations. Interestingly, at these concentrations, E4 partially antagonized the pro-tumoral effect of E2.

### E4 does not antagonize suppress the nuclear ERα actions

**Figure 7:**
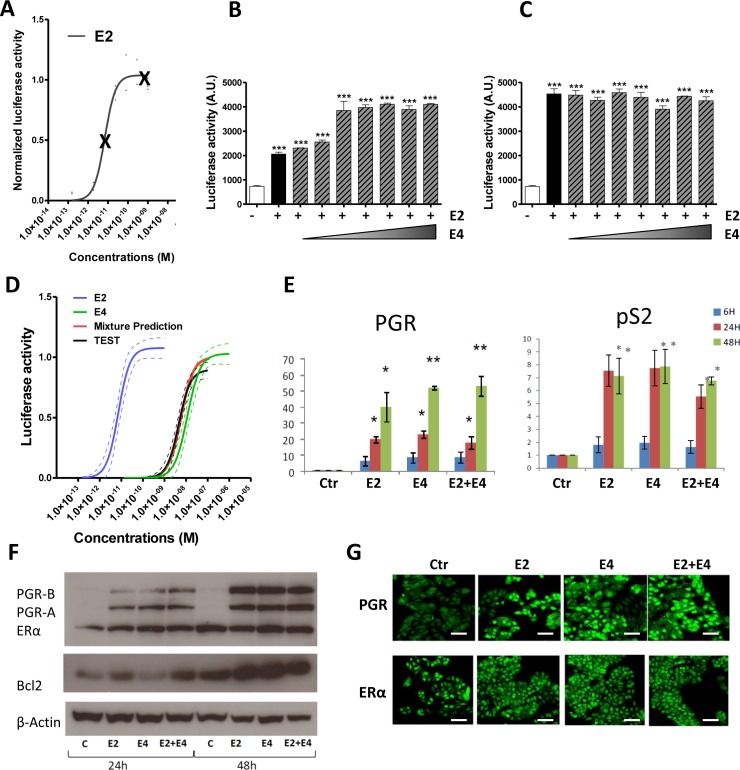
Impact of the combination E2 + E4 **A.**-**C.**, effect of the combination E2 + E4 in the ERE-luciferase reporter activity. T47D-KBluc cells were treated with two fixed concentrations of E2 (A): 1×10^−11^M (B) or 1×10^−9^M (C) in combination with increasing concentrations of E4 (from 1×10^−11^ to 1×10^−5^M). D, Mixture effect prediction on the ERE-luciferase reporter activity. Prediction curve (red) was calculated from separate E2 (blue) and E4 (green) curves using dose addition model. The prediction was then tested experimentally (TEST curve, black). Graph shows regression curves and their 95% confidence belts (dotted lines). **E.**, quantitative RT-PCR analysis of *PGR* and *PS2* genes from MCF-7 cells treated with E2 1×10^−8^M, E4 1×10^−6^M or vehicle (ctr) during 6, 24 or 48hours. **F.**, representative western blot of PGR, ERα, BCL2 and β-actine after treatment with E2 1×10^−8^M, E4 1×10^−6^M or E2+E4 during 24 or 48 hours. **G.**, immonufluochemistry of PGR and ERα in MCF-7 cells after treatment with vehicle (ctr), E2 1×10^−8^M, E4 1×10^−6^M or E2+E4 during 48 hours. Scale bar = 50μm. *, *P* ≤ 0.05; **, *P* ≤ 0.01; ***, *P* ≤ 0.001 versus control group.

We demonstrated above that E4 induces both nuclear and extra-nuclear signaling, albeit less efficiently than E2. Thus, trying to understand the mechanisms underlying the antagonistic effects of E4 on E2, we assessed the impact of E2+E4 combination on ERE-dependent activity of ERα. Increasing concentrations of E4 were combined with two different concentrations of E2 (1×10^−11^M and 1×10^−9^M) inducing submaximal and maximal effects, respectively (Figure 7A). In these conditions, E4 failed to antagonize the effects of E2 on ERE-Luc transactivation induction in T47D-KBluc cells. On the contrary, a cumulative effect of both compounds was observed when E2 was used at the submaximal concentration to reach maximal ERE activity (Figure 7B). No effect of E4 was observed on luciferase activity when E2 was used at a dose that already induced maximal ERE activity (Figure 7C). Same results were obtained using ERE-Luc transfected MCF-7 cells line (Suppl. Figure 3).

Further experiments were conducted by predicting and testing the effect of a binary mixture between E2 and E4. The prediction was calculated with dose addition, a commonly used concept in toxicology for the prediction of chemical mixture additive effects [38]. The prediction was then tested experimentally. Data showed that the predicted combination effect agreed very well with the experimentally observed data (Figure 7D) confirming that E2 and E4 do not have any antagonistic effect on ERα binding to ERE when they are combined. Moreover, the combination of E2+E4 failed to modulate the E2-induced expression of genes harboring ERE in their promoter region, such as *pS2*, *BCL2* and *PGR* at mRNA and/or protein level (Figure 7E-7G).

These data clearly demonstrate that E4 does not antagonize the nuclear actions induced by E2.

## DISCUSSION

The identification of new estrogenic compounds for MHT that selectively preserve the beneficial effects of estrogens while reducing their unwanted side effects, such as breast cancer promotion, is largely needed. Several evidences have suggested that E4 could act as a SERM [9-16]. However, its impact on breast cancer remains controversial and poorly understood since it is reported to be either an estrogen or an anti-estrogen. The preclinical results presented in this study indicate a dose-dependent dual weak estrogenic/anti-estrogenic activity of E4 on breast cancer and delineate a safe therapeutic window for menopause treatment.

Below 10 nM, E4 does not increase breast cancer growth, while E2 already causes a maximal stimulation. On the other hand, when used at concentrations higher than 10 nM, E4 exhibits an estrogenic activity by promoting breast cancer cell growth in line with previous *in vitro* studies [19, 20]. It is important to consider that doses of E4 necessary to elicit such estrogenic effect on malignant breast cells are high pharmacological doses that would not be used for therapeutic purposes. Interestingly, E4 also presents an antitumor activity by reducing the strong mitogenic effect induced by E2, with a maximal antagonist effect seen when the concentration ratio E4/E2 is equal to 100. Our findings can contribute to explain that high doses of E4 suppress mammary tumors induced by DMBA in rats [18]. This unique dual activity of E4 observed on breast cancer is also in line with promising data we previously reported on the reduced impact of E4 on normal mammary gland proliferation [17].

Preclinical studies have demonstrated that 0.3mg E4/kg/day abolishes hot flushes in a rat model [11] and that 0.5mg E4/kg/day elicits bone-sparing effects [12]. Moreover, in women, a daily oral dose of 2 mg E4 (~ 0.03mg/kg/day) has an estrogenic effect on vaginal cytology while having a neutral protective effect on endometrial proliferation (Foidart & Coelingh Bennink, congress of International Menopause Society 2014, personal communication). Notably, in our experimental conditions, these E4 doses failed to promote breast tumor growth. While the stimulation of breast cancer growth by E4 requires doses 100 to 1000-fold higher than E2, E4 doses that are only 2-4 times higher than E2 abrogate efficiently the clinical symptoms of menopause in other target tissues (brain and vaginal keratinocytes). These data clearly highlight that E4 could be effective in women for the relief of menopausal symptoms at doses associated with no or limited impact on the breast, suggesting a large window of therapeutic safety.

The properties of E4 concerning cell proliferation and apoptosis pointed out in this work are in line with previous studies showing that estrogens promote breast cancer not only by stimulating cell proliferation but also by impacting the apoptotic pathways [30]. Nevertheless, our results diverge from the observations of Singer [21] reporting an increase of apoptosis in tumor of women treated during 14 days with 20mg E4 (~ 0.3mg/kg/day). As pointed out by the authors, this study was a small exploratory trial in which immunohistochemical analyses were conducted on a limited number of breast cancer samples. In contrast to E2, E4 is not cytotoxic even at concentrations higher than 1×10^−6^M, suggestive of a good tolerability. This observation is in agreement with the high E4 concentrations naturally found in the amniotic fluid and in the fetal and maternal plasma during pregnancy rising to 60 nM and evidently safe for the fetus and the mother [39].

Our results clearly demonstrate that ERα is necessary to drive the mitogenic effect of E4 on breast cancer growth. Since 70 % of breast cancers are diagnosed positive for ERα, this observation further underlines the crucial need to precisely characterize the impact of E4 on breast cancer before any clinical use for MHT. The contribution of GPER to estrogen-induced signaling in breast cancer remains a matter of debate. Some studies report a contribution of GPER in the signaling induced by E2 [40] and in the proliferation of normal and malignant breast [41], although other reports failed to demonstrate this kind of observations [42]. In our experimental conditions, we observed that GPER was not sufficient to induce breast cancer cell growth. However, preliminary evidences suggest that it could partially contribute to this effect and to E4-induced signaling. One possibility is that E4 binds and activates ERα, and this communicates to membrane localized GPER. This specific controversial issue oversteps the purpose of this study and will have to be addressed with appropriates tools since the possible involvement of GPER in E4 signaling suggested here, only relies on the specificity of G15 as a GPER antagonist.

Mechanistically, we demonstrate that E4 induces both nuclear and extra-nuclear signaling pathways. This agonistic profile of E4 on nuclear ERα activation observed in breast cancer is consistent with previous *in vivo* experiments performed on three recognized nuclear ERα-dependent processes showing that high doses of E4 promote uterine gene expression, endometrial proliferation and prevention of atheroma [13]. The antagonistic activity exerted by E4 on E2-dependent breast cancer growth is clearly not mediated by a modulation of these ERα nuclear actions. This corroborates the observation that E4 fails to antagonize E2-dependent uterotrophic effects driven by ERα nuclear actions [13]. In contrast, E4 antagonizes E2 extra-nuclear effects in the endothelium resulting in an inhibition of eNOS phosphorylation and NO release [13]. In breast cancer cells, we previously reported that a 5 minutes stimulation with either E2 or E4 significantly stimulated the cytoplasmic interaction between ERα and Src. Rather surprisingly, the combination E2+E4 had no stimulatory effect on this interaction [13]. In addition, E4 decreases the E2-induced moesin phosphorylation, leading to a subsequent reduction of cell migration [22]. The molecular mechanisms driving extra-nuclear actions of estrogens are far from being fully understood. The downstream targets regulated by the membrane-associated form of ERα involve various post-transcriptional modifications that highly differ between cell types. In addition, the number and variety of signaling proteins involved convey specificity to the estrogen signaling in a cell- and context-dependent fashion and result in differential cell activities [26]. Thus, it is important to consider a possible involvement of GPER and other signaling molecules in modulating E4 action on malignant breast cells.

In summary, a major issue to improve postmenopausal women's health is to identify new compounds that present an estrogenic effect on the vagina, the bone, cardiovascular and central nervous systems, while having minimal impact on the breast. The exclusive dual weak estrogenic/anti-estrogenic activity of E4, through tissue-specific crosstalk between nuclear and extra-nuclear signaling pathways, opens promising possibilities. There is, of course, a large frontier between preclinical mechanistic studies and clinical performance of any new drug. Therefore, clinical studies are now needed to confirm the efficacy and the safety of E4 to relieve menopausal symptoms.

## MATERIALS AND METHODS

### Chemicals, drugs and antibodies

17β-estradiol (E2), epidermal growth factor (EGF), tamoxifen and fulvestrant (ICI 182,780) were purchased from Sigma-Aldrich (St-Louis, MO, USA). G15 (GPER-antagonist) was obtained from Cayman Chemical Company (Ann Arbor, MI, USA). E4 was supplied by Uteron Pharma (Liège, Belgium). All compounds were dissolved in ethanol (EtOH). Dulbecco's Modified Eagle medium (DMEM), Roswell Park Memorial Institute medium (RPMI), fetal bovine serum (FBS), dextran-coated charcoal treated-FBS, MEM-non-essential amino acid, glutamate, penicillin and streptomycin were purchased from Invitrogen (Carlsbad, CA, USA). ERα and GPER antibodies were purchased from Santa-Cruz Biotechnology (Santa Cruz, CA, USA). ERβ and PGR antibodies were from Novocastra (Wetslar, Germany). BCL2 antibody was from Dako (Trappes, France). Antibodies for phospho-ERα, phospho-ERK1/2, total ERK1/2 and phospho-AKT were obtained from Cell Signaling Technology (Beverly, MA, USA).

### Cell culture

Human breast cancer cells (MCF-7, T47D, MDA-MB-231 and SKBR3) were purchased and authenticated from ATCC. All cell lines were authenticated within 1 year before being used in experiments. Cells were routinely cultured in DMEM supplemented with 10% FBS, L-glutamine (2 mmol/L), penicillin (100 IU/mL) and streptomycin (100μg/mL) at 37°C in a 5% CO_2_ humid atmosphere. 24 h before the experiments with steroids, the medium was removed and replaced with DMEM without phenol red supplemented with dextran-coated charcoal-treated FBS to exclude estrogenic effects caused by the medium or the serum.

### Cell growth assay and cell proliferation assay

Cells were seeded in 96-well culture plates at a density of 5,000 cells/well and then incubated for 72 h with different concentrations of E4 or E2. Cell growth was measured by the Cyquant Kit from Invitrogen (Carlsbad, CA, USA) following the manufacturer's instructions, dosing the total quantity of DNA in a well. The amount of fluorescence was measured on a plate reader at a wavelength of excitation/emission 480/520 nm.

Cell proliferation was measured using thymidine incorporation. 24 h after the beginning of hormonal treatment, cells were incubated with 2 μCi of methyl-^3^[H] thymidine for 48 h at 37°C. After incubation, cells were washed and incubated in 5% Trichloroacetic acid for 15 min at 4°C and lysed in NAOH 0.1M for 15 min at 37°C. The total lysate was added to 25 μl of scintillation liquid and radioactivity was counted with a β-counter (Beckman, LS-5000-CE).

### E-screen assay

MCF-7 BOS cells are highly estrogen-responsive breast cancer cells and for this reason, considered as the most appropriate cell line for the E-Screen [43]. MCF-7 BOS cells were routinely maintained in DMEM with Glutamax supplemented with 5% FBS and 1% MEM-non-essential amino acids. The assay was conducted as described previously [43]. Briefly, cells were seeded into 96-well plates at a density of 2,500 cells per well and allowed to attach for 24h. The seeding media was then replaced with the experimental medium consisting of phenol-red free DMEM supplemented with 1% sodium pyruvate, 1% MEM-NEAA and 10% CD-FBS. Each plate contained 1 row (8 wells) negative controls (0,5% EtOH), 1 row positive controls (saturating concentration E2) and 8 increasing concentrations of the test chemical, tested in duplicate. Following 120 h incubation, the assay was terminated and the cells were fixed with a 10% solution of ice cold trichloroacetic acid for 25 min. The plates were then washed with water, allowed to air dry and stained with 0.4% sulforhodamine B in 1% acetic acid for 10 min. Unbound dye was completely removed by rinsing with 1% acetic acid and bound SRB was solubilized with 10mM Tris. The optical density (O.D.) was read at 510 nm directly in the same plate on a microplate reader (Labsystems Multiskan, UK).

### Cell death assay

MCF-7 cells were seeded in 96-well plates (5,000 cells/well) and left to adhere overnight. Cells were then treated with indicated compound and concentration. After 72 h of treatment, apoptosis level (histone-associated DNA fragments) was determined using the Cell Death Detection Elisa from Roche (Bâle, Switzerland) following the protocol provided by the manufacturer. Briefly, the cytoplasmic fractions were added to the 96-well ELISA plates pre-coated with the anti-histone monoclonal antibody and incubated for 2 h at room temperature. After washing, bound nucleosomes were detected by the addition of anti–DNA-peroxidase monoclonal antibody and reacted for 1h at room temperature. After the addition of substrate, the optical density was read with a microplate reader at 405 nm. Values were normalized to ADN content in each well.

### Cytotoxicity assay

The cell cytotoxicity was determined after 72 h of treatment using the ApoTox-Glo Triplex Assay kit from Promega (Fitchburg, WI, USA) following the manufacturer's protocol. Fluorescent signal was determined using the plate reader Victor II-PerkinElmer. Values were normalized to ADN content in each well.

### Luciferase gene reporter assay

The ER-LUC assay was performed as previously described [44]. Briefly, for seven days prior to experiments, T47D-KBluc cells were maintained in low estrogen conditions by the use of pre-assay media (RPMI, 10% charcoal-dextran stripped FCS, no antibiotics). For experiments, cells were seeded in white 96-well plates at a density of 10,000 cells/well and allowed to attach for 24h before removal of media, and application of test chemicals in dosing media (phenol red-free RPMI, 5% charcoal-dextran stripped FCS, no antibiotics). The positive control was 1 nM E2. 24h after application of test and control solutions, a volume of Steady-Glo assay reagent from Promega (Fitchburg, USA) equal to the volume of culture media was added to allow the cell lysis. Plates were then loaded into a plate reader followed by measurement of luminescence (FLUOstar Optima, BMG Labtech GmbH).

### The recombinant yeast estrogen screen

The assay was carried out exactly as described previously [45]. Briefly, growth media was inoculated with yeast stock and grown overnight in an orbital shaker at 28°C. The assay medium consisted of 50 ml of growth medium, chlorophenol red-ß-D-galactopyranoside (10 mg/l) and 2 ml of the overnight yeast culture. Aliquots of 10 μl of the ethanolic dilutions of E2 or E4 were transferred to 96-well plates and allowed to evaporate to dryness. Final tested concentrations of E2 and E4 ranged between 1×10^−12^ and 1×10^−8^, and 1×10^−8^ and 1×10^−4^ M, respectively. All plates included a row of EtOH controls (i.e. no test agent) and a row of assay medium without yeast cells (blanks). To each well, except the blanks, a volume of 200 μl of yeast-seeded assay medium was added. Plates were sealed and shaken vigorously for 2 min before incubating at 32 °C, in a humidified box for 72h. Plates were then analyzed spectrophotometrically at 540 nm (color) and 620 nm (turbidity) using a Labsystem Multiskan Multisoft plate reader.

### Western blot analysis

Cell were exposed to test chemicals during appropriate time, and then lysed in lysis buffer containing a protease inhibitor. 20 μg of whole cell total proteins were separated onto SDS-polyacrylamide gels and transferred to nitrocellulose membrane. Membrane was blocked with 5% milk/PBS/Tween and incubated with primary antibodies overnight at 4°C. Membranes were then washed and incubated with appropriate HRP-conjugated secondary antibodies and detected using an ECL system. β-actin was used as a control for equal loading and total protein in case of phosphorylated protein. Results of densitometry analyses of western blots, obtained using QuantityOne software from Biorad (Hercules, CA, USA), are presented as optical densities relative to the control.

### Quantitative real-time PCR

Total RNA was extracted from cells using Trizol Reagent from Invitrogen (Carlsbad, CA, USA) according to the manufacturer's protocol. The cDNA was synthesized with 2 μg of total RNA using random primers for 1h at 37°C. Real-time quantitative PCR was performed using specific primers and Brilliant SYBR GREEN QPCR master mix from Qiagen (Hilden, Germany). GAPDH was amplified as an internal control. Sequence primers for target genes are reported in Table 1.

**Table 1:** Primer sequences used for quantitative RT-PCR

Name	Primer
*GAPDH*	FORWARD TGCTGTAGCCAAATTCGTTG REVERSE ACCAGGTGGTCTCCTCTGAC
*Cyclin D1*	FORWARD GCTGTGCATCTACACCGACA REVERSE TTGAGCTTGTTCACCAGGAG
*BRCA1*	FORWARD TTGTTGATGTGGAGGAGCAA REVERSE GATTCCAGGTAAGGGGTTCC
*BAD*	FORWARD CGAGTTTGTGGACTCCTTTAAGA REVERSE CACCAGGACTGGAAGACTCG
*BCL2*	FORWARD ACAGAGGATCATGCTGTACTTAAAAA REVERSE TTATTTCATGAGGCACGTTATTATTAG
*PGR*	FORWARD AGCCCACAATACAGCTTCGAG REVERSE CCAGCCTGACAGCACTTTCT
*PS2*	FORWARD GCCCAGACAGAGACGTGTACAGT REVERSE CTGGAGGGACGTCGATGGTATTAG

### Immunofluorescence staining

Cells were grown on coverslips. After treatment, cells were fixed 10 minutes at room temperature with paraformaldehyde 4%- HEPES 250nM. Cells were permeabilized 30 minutes at room temperature with PBS-Triton 1%. After saturation of non-specific sites with 1% PBS-BSA, cells were then incubated 2 hours at 37°C with progesterone receptor primary antibody. Cells were subsequently incubated for 20 minutes at 37°C with the fluorescent Alexa Fluor 488 conjugated IgG secondary antibody. Then, cells were stained with DAPI and the coverslips were mounted on slides with the Prolog Gold reagent from Invitrogen (Carlsbad, CA, USA).

### *In vivo* experiments

Immunodeficient mice were ovariectomized to prevent endogenous estrogen production. MCF-7 cells (2×10^6^ cells suspended in 400 μl Matrigel) were injected subcutaneously into each flank of mice. Mice received then a daily oral administration of vehicle (peanut oil + 5% EtOH), E2 (3mg/kg/day) or E4 (0.5 to 10 mg/kg/day). Mice were sacrificed and tumor weighed after 5 weeks of treatment. For combined treatment of E2+E4, mice were implanted subcutaneously with a pellet releasing E2 (1.7 mg, Innovative Research of America). When tumors were palpable and reached an average area of 40-50mm^2^, animals were divided into several groups (5 animals /group). E2 pellets were removed from mice of the control untreated group. Other groups received a fresh E2-releasing pellet and a daily oral treatment of vehicle (peanut oil + 5% EtOH) or E4 (1, 3 and 10 mg/kg/day). Tumors were measured every week with digital caliper and tumor volume was calculated as V (mm^3^) = π × [(width)^2^ × length]/6. After 5 weeks of treatment, mice were sacrificed. We systematically checked that untreated ovariectomized mice had an atrophied uterus (<10 mg) and that mice implanted with an E2-releasing pellet had a significant increase of uterine weight. All animal procedures were performed according to the Federation of European Laboratory Animal Sciences Associations within the accredited GIGA animal facility (University of Liège).

### Histological analysis

To carry out histological analysis, tumor samples were fixed in 4% formalin for 4h and stored in 70% EtOH before paraffin embedding. The sections were cut at 6 μm. For detection of pERK and pAKT, the slides were deparaffinized in xylene and rehydrated through graded alcohols. For antigen retrieval, sections were heated in 10 mM citrate buffer for 10 min. The sections were then treated with 3% H_2_O_2_ for 20 min to block endogenous peroxidase activity, washed with PBS, and incubated with 10% BSA for 1h. After blocking, the sections were incubated with the pERK antibody and pAKT antibody at 1:100, and 1:250 dilutions respectively. The slides were then incubated with biotinylated secondary antibodies for 30 min, followed by 30 min incubation with streptavidin–peroxidase conjugate. Antigen– antibody complex was visualized by incubation with 3,30-diaminobenzidine 5. The slides were counterstained with hematoxylin, dehydrated, and mounted using a mounting medium from Labonord (Templemars, France). Positively stained cells appeared brown.

### Preparation of mixtures and calculation of mixture effect predictions using dose addition model

Dose addition (DA) is a widely used pharmacological concept for the prediction of chemical mixture effects when only the effect of individual components is known. This hypothesis expresses the expected combination effect based on the assumption that all mixture components exert their effects without influencing each other's action. Using the DA additivity prediction, it is then possible to assess experimentally observed mixture effects in terms of synergisms or antagonisms [38]. Here, the mixture experiment was designed according to the fixed mixture ratio design, where serial dilutions of a stock solution of a mixture of E4 and E2 were made and then tested.

The mathematical and statistical procedures used for calculating DA mixture effects are described in [46]. Differences between predicted and observed effects were deemed statistically significant when the prediction did not overlap with the 95% confidence belts of the experimentally observed mixture effects.

### Statistical analysis

All quantitative experimental data are expressed as mean ± SEM. Statistical analysis were conducted with GraphPad Prism 4.0 software (La Jolla, CA, USA) using one-way ANOVA followed by Student-Newman-Keuls's test or using Kruskal-Wallis followed by Dunn's test, with regard to heterosedasticity. The value of *P* ≤ 0.05 was considered as statistically significant.

## SUPPLEMENTARY MATERIAL FIGURES


