# Supplementary material for: Combined estrogenic and anti-estrogenic properties of estetrol on breast cancer may provide a safe therapeutic window for the treatment of menopausal symptoms
Source: Oncotarget. 2015 May 19;6(19):17621–36. doi: 10.18632/oncotarget.4184 (PMC4627333; doi:10.18632/oncotarget.4184)
Supplement: Supplementary file 1 [file oncotarget-06-17621-s001.pdf]

Combined estrogenic and anti-estrogenic properties of estetrol on breast cancer may provide a safe therapeutic window for the treatment of menopausal symptoms

Supplementary Material

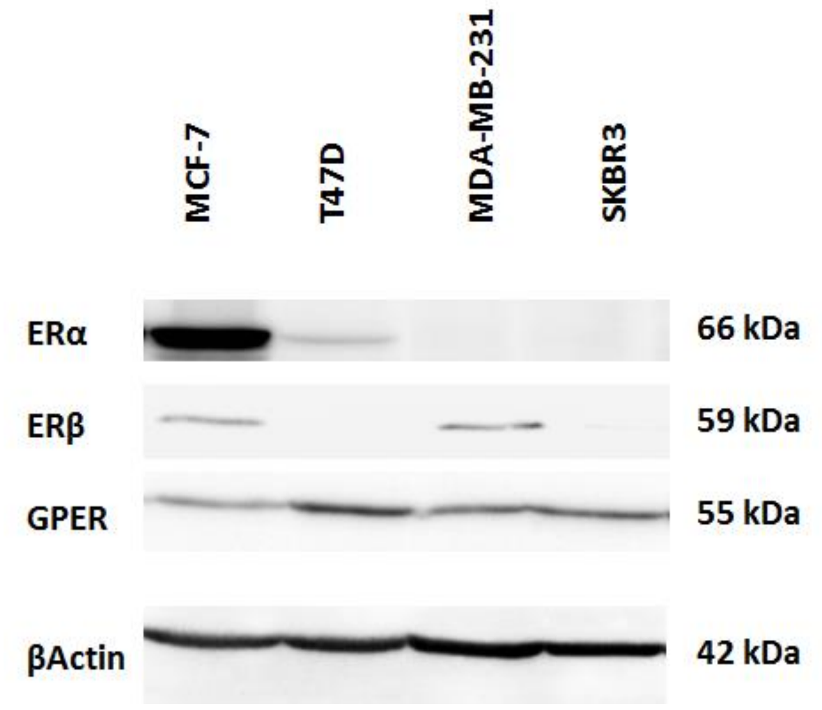

<u>Suppl Figure 1</u>

Estrogen receptor status in MCF-7, T47D, MDA-MB-231 and SKBR3 cells.
ERα, ERβ and GPER expression detected by immunoblotting.

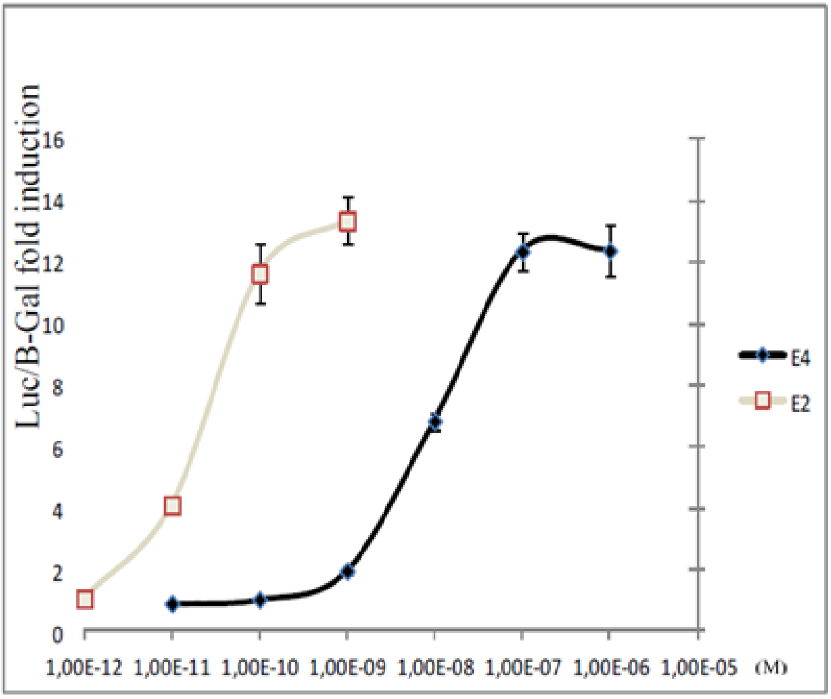

Suppl Figure 2

Transactivation of an ERE-Luc reporter gene.

MCF-7 cells were transfected with the ERE-Luciferase reporter and treated with increasing concentrations of E2 (grey curve) and E4 (black curve). Results are expressed as mean ± SEM fold induction for the Luciferase normalized by β-galactosidase activation.

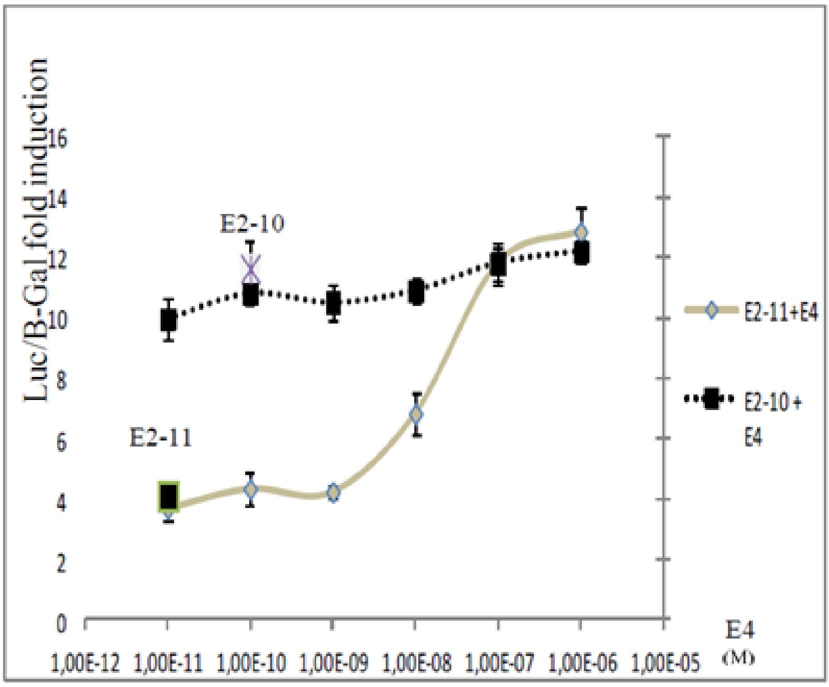

Suppl Figure 3

Transactivation of an ERE-Luc reporter gene: impact of E2+E4.

MCF-7 cells were transfected with the ERE-Luciferase reporter and treated with different combinations of E2+E4. E4 was added at increasing concentrations in the presence of E2 at 2 different concentrations ($1x10^{-11}$M or $1x10^{-10}$M). Results are expressed as mean ± SEM fold induction for the Luciferase normalized by β-galactosidase activation.
